# Collectanea Medica: Consisting of Anecdotes, Facts, Extracts, Illustrations, &c.

**Published:** 1821-08

**Authors:** 


### COLLECTANEA MEDICA:

CONSISTING OF

### ANECDOTES, FACTS, EXTRACTS, ILLUSTRATIONS, &c.

*Relating to the History or the Art of Medicine, and the Auxiliary Sciences.*

Floriferis ut apes in saltibus omnia libant,

Omnia nos itidem depascimur aurea dicta.

---

### *Extracts of the Report from the Select Committee of the House of Commons on the Doctrine of the Contagion of the Plague, in* 1819.

*Jovis*, 13° *die Maij*, 1819.

SIR JOHN JACKSON, Baronet, in the Chair.

RICHARD HARRISON, M.D. called in; and Examined.

HAVE you had any opportunity of seeing the plague?—I was at Naples in 1816 and 1817, during part of that time the plague raged severely at Noja, a town situated about 150 miles from that place; and I have had frequent conversations on the subject with the medical men who had the direction of the sick during that time. I saw, also, at Naples, in the spring and summer of 1817, several persons labouring under a contagious disease, which corresponded with the descriptions generally given of the plague; but will not assert it to have been that disorder. The mortality from it was so great, that our consul was in doubt whether it would not be right to give public notice of the circumstance.

Describe the appearances of what you consider to be plague?—I consider the plague as a typhous fever, with the ordinary symptoms more severe; and, in addition, broad purple spots, carbuncles, buboes of the parotids, but more generally in the arm-pits or groin.

Do you consider the plague as contagious?—Assuredly.

And the typhous fever also?—Yes; assuredly.

You consider the plague as a high degree of typhus, and coming under the same classification?—The two diseases may probably arise from different causes, but they frequently so nearly resemble each other, differing only apparently in degree, that I think it possible they may be produced from the same cause. I would not be understood to say positively that I consider them as the same disease.

You are not sure that you have seen the true plague?—No: I saw some cases at Naples labouring under the symptoms I have before mentioned.

If a case of that kind had occurred at Constantinople, would it not have been considered as decisive of the plague?—I should conceive it would.

What do you suppose to be the cause of the plague?—Contagion, I should think.

From the revival of former virus, or the occurrence of another originating cause?—I conceive it may be propagated in both ways. The causes which first produced it might produce it again, although it had entirely disappeared; or the disease constantly existing may be of so mild a character as to escape attention, awaiting favourable circumstances to render it active.

You consider that local causes alone might revive it annually?—I should think very probably, under favourable circumstances, in the Levant; but in Europe the disease is only spread by contagion, for the separation of the healthy invariably preserves them.

Do you consider it very similar to small-pox contagion?—I cannot draw a comparison; I consider the two diseases as so different.

Is the contagious quality of it so likely to communicate the disease as small-pox?—I can conceive the plague existing, and yet not very contagious; and I can conceive it existing and extremely contagious; and precisely the same with the small-pox.

Do you not consider the small-pox contagion as more diffusible than the plague?—I should think it difficult to institute the comparison.

To what do you attribute our not having had any plague-case in Great Britain for so long a period? Having had notice only last night, I have scarcely had time to give it a thought. I conceive, when a cargo is shipped in any part of the Levant, the sailors of the ship are necessarily employed in taking in the cargo. Under these circumstances, if the plague existed, I do not suppose the ship would arrive in England before the disease had made its appearance; and I suppose, when the plague breaks out on the voyage, the vessels put into the nearest port where quarantine can be performed, and are there subjected to the usual precautions. This may be only one reason among others.

Do you consider goods equally communicable of the disease as persons?—From all I have heard, inquiring of those who have seen the plague very repeatedly, and what I have read, I conceive equally.

How do you account for the expurgatórs of goods in Great Britain at the quarantine establishments never having taken the plague?—I should suppose the plague makes its appearance before the ships arrive in England, and I conceive the goods liable to give the plague have undergone purification in foreign lazarettos before their importation into England; and we frequently find a typhous fever, probably from this cause, in a lazaretto: of this I had examples during my attendance on the sick in the quarantine establishment at Naples.

Do you know that the goods are promiscuously shipped at Smyrna, whether or not the plague rages there?—With that I am not acquainted.

Assuming that to be the fact, how do you account for those goods, upon their arrival in Great Britain, not communicating the disease at the quarantine establishments?—I should suppose the plague makes its appearance before the ship arrives, and consequently the necessary precautions have been employed.

The fact being that goods are shipped at Smyrna, even in times when the plague rages furiously, and those goods arriving at the quarantine establishment in Great Britain, how do you, in those cases, account for the expurgators of goods not having the infection?—Although we have authentic accounts of the plague existing at nearly every temperature, still we find it is less virulent at one temperature than at another: perhaps other causes may conspire with temperature to increase its destructive effects. The degree of temperature, and other favourable circumstances for the propagation of plague, not existing at the time the affected goods arrived in England, may have prevented the dissemination of the disorder. But, should such circumstances exist, we may again be visited by the plague, and even a typhous fever may have been produced in this country by such goods; for we know that a mild case of plague resembles much typhous fever.

It appears from the Custom-house return, that nothing of that kind has appeared at the quarantine establishments ever since their origin: from that circumstance would you not conclude the infection did not arrive?—To give a satisfactory answer to that question would require more reflection than I have been able to give to this subject.

Do you consider the plague of 1665 to have been imported into Great Britain?—I feel unequal to answer that question.

Do you consider the quarantine establishments as useful?—As very useful: but I should conceive, from what I have seen in the quarantine establishments here, at Venice, Naples, Malta, and other ports of the Mediterranean, that considerable modifications might take place: amongst many other examples, I might instance a circumstance which occurred to myself. I arrived at Dover from Naples in seventeen days, but neither myself, luggage, or carriage, underwent any-where quarantine; whereas my other luggage, which I left at the same time, came by sea, and arrived in England a considerable time after me, underwent the usual quarantine. Couriers, who travel with the greatest expedition, do not perform quarantine; although ships, which have taken a long time to perform the voyage from the same place, are frequently obliged to undergo it.

You consider that the regulations might be modified, both with respect to persons and goods?—With respect to goods, I am unable to give an opinion; but I should assuredly think so with respect to persons.

---

[The extracts already produced from the Minutes of Evidence comprise the examination of the whole of the Physicians who have had opportunities for actual observation: we shall conclude with transcribing the *General Report of the Committee.*]

The Select Committee appointed to consider the validity of the doctrine of Contagion in the Plague; and to report their observations thereupon, together with the minutes of the evidence taken before them, to the House;—have considered the matters to them referred, and have agreed upon the following REPORT:

Your Committee being appointed to consider the validity of the received doctrines concerning the nature of contagious and infectious diseases, as distinguished from other epidemics, have proceeded to examine a number of medical gentlemen, whose practical experience or general knowledge of the subject appeared to your Committee most likely to furnish the means of acquiring the most satisfactory information. They have also had the evidence of a number of persons whose residence in infected countries, or whose commercial or official employments, enabled them to communicate information as to facts, and on the principle and efficacy of the laws of quarantine. All the opinions of the medical men whom your Committee have examined, with the exception of two, are in favour of the received doctrine, that the plague is a disease communicable by contact only, and different in that respect from epidemic fever; nor do your Committee see any thing in the rest of the evidence they have collected, which would induce them to dissent from that opinion. It appears from some of the evidence, that the extension and virulence of the disorder is considerably modified by atmospheric influence; and a doubt has prevailed whether, under any circumstance, the disease could be received and propagated in the climate of Britain. No fact whatever has been stated to show that any instance of the disorder has occurred, or that it has ever been known to have been brought into the lazarettos for many years: but your Committee do not think themselves warranted to infer from thence that the disease cannot exist in England; because, in the first place, a disease resembling in most respects the plague is well known to have prevailed here in many periods of our history, particularly in 1665-6; and further, it appears that, in many places and in climates of various nature, the plague has prevailed after intervals of very considerable duration.

Your Committee would also observe, down to the year 1800, regulations were adopted which must have had the effect of preventing goods infected with the plague from being shipped directly for Britain; and they abstain from giving any opinion on the nature and application of the quarantine regulations, as not falling within the scope of inquiry to which they have been directed; but they see no reason to question the validity of the principles on which such regulations appear to have been adopted.

*14th June*, 1819.

